# Proximal Fibular Osteotomy in the Management of Osteoarthritis of Medial Compartment of Knee Joint

**DOI:** 10.7759/cureus.8481

**Published:** 2020-06-06

**Authors:** Masroor Ahmed, Muhammad Bux, Mukesh Kumar, Naveed Ahmed, Ghulam Hussain, Muhammad Ishtiyaque

**Affiliations:** 1 Orthopedic Surgery, United Medical and Dental College, Karachi, PAK; 2 Orthopedic Surgery, Shaheed Muhatarma Benazir Bhutto Medical College Lyari, Karachi, PAK; 3 Orthopedic Surgery, Awadh General Hospital, Karachi, PAK; 4 Orthopedic Surgery, Khairpur Medical College, Sindh, PAK; 5 Orthopedic Surgery, Kulsoom Bai Valika Social Security Hospital, Karachi, PAK; 6 Orthopedic Surgery, Sindh Government Qatar Hospital, Karachi, PAK

**Keywords:** proximal fibula, osteotomy, medial compartment, osteoarthritis

## Abstract

Introduction: This study was conducted to evaluate the efficacy of proximal fibular osteotomy (PFO) in terms of pain relief, and improvement in function in medial compartment osteoarthritis (OA) of the knee joint.

Materials and methods: This case series study was conducted at the Shaheed Mohtarma Benazir Bhutto Medical College Lyari and United Medical and Dental College of Karachi. Patients with medial compartment knee joint OA were included in the study and patients with bicompartmental or tricompartmental OA, inflammatory joint disease, valgus knee deformity, morbid obesity, or any infectious pathology involving the knee joint were excluded from the study. The medial and lateral joint spaces were measured and recorded preoperatively and postoperatively. Pre- and postoperative values of visual analog scale (VAS) results and the Oxford knee score were recorded. A PFO was performed after getting informed written consent.

Results: A total of 60 patients were enrolled in the study; 16 (26.7%) were men, and 44 (73.3%) were women. The mean age of patients was 51.8 ± 4.1 years. The mean preoperative medial joint space measurement on standard anteroposterior radiograph was 1.45 ± 0.28 mm. The mean preoperative lateral joint space was 8.86 ± 1.27 mm. The recorded mean preoperative Oxford knee score was 20.82 ± 1.97 mm. Recorded levels of mean postoperative medial joint space improved to 4.63 ± 0.668 mm, and mean postoperative lateral joint space was 4.72 ± 0.79 mm. Mean recorded levels of VAS for pain postoperatively were 2.32 ± 0.792, which improved significantly from 7.90 ± 0.79.

Conclusions: PFO is a good surgical technique for pain relief and functional improvement in patients suffering from medial compartment OA.

## Introduction

Osteoarthritis (OA) of the knee joint is a chronic, degenerative problem often associated with pain involving the affected joint, decreased range of motion, and deformity [1]. Symptomatic OA affected about 3.8% of the world population according to estimates in 2010, with higher involvement in women (4.8%) as compared to men (2.8%) [2]. OA of the knee joint affects about half of the population over the age of 60 years and mainly women, as it is mostly because of osteoporosis as a result of decreased bone mineral density [3]. There are multiple options for the management of knee joint OA, both conservative and surgical. Conservative options for OA of the knee include analgesics, physical therapy, intra-articular injections of steroid or platelet-rich plasma, and viscosupplementation agents [4-7]. Surgical options include high tibial osteotomy and total knee arthroplasty, the main treatment options for OA of the knee [8]. High tibial osteotomy is a technically demanding procedure and has specific problems associated with it, such as neurovascular injury, iatrogenic fracture, and nonunion [9-10]. Total knee replacement is an excellent procedure in terms of relief of pain, correction of deformity, and improvement of function but not a good option for young patients with mild to moderate OA [11]. The medial compartment is most commonly involved in knee joint OA because, in healthy people, it carries almost 60%-80% of the load as the mechanical axis lies more frequently medial to the center of the knee joint [12]. Proximal fibular osteotomy (PFO) is an excellent treatment option for medial compartment OA in comparison to high tibial osteotomy [1], as it is associated with fewer or none of the complications [13] that are frequently encountered in a high tibial osteotomy. PFO works on the principle that it supports the lateral tibial plateau and by removing the wedge from the fibula, it weakens the support of lateral tibial plateau provided by the fibula leading to correction of Varus deformity, shifting the loading force from the medial compartment more laterally causing the pain to decrease and improvement in function [1]. When comparing a high tibial osteotomy to a proximal, it is a relatively simple surgical procedure. The surgical indication for PFO is medial compartment knee joint OA [1].

This study was conducted to evaluate the efficacy of PFO in terms of pain relief and improvement in function.

## Materials and methods

This descriptive case series study was conducted at the Shaheed Muhatarma Benazir Bhutto Medical College Lyari and United Medical and Dental College of Karachi from 15 June 2016 to 15 June 2018. Patients with medial compartment knee joint OA were included in the study whereas patients with bicompartmental or tricompartmental OA, inflammatory joint disease, valgus knee deformity, morbid obesity, or any infectious pathology involving the knee joint were excluded from the study.

The study was approved by the ethical review committee. For all the patients who were enrolled for study, a clinical and radiological evaluation was performed. A weight-bearing radiograph of the affected knee was performed in anteroposterior and lateral views, and medial and lateral joint spaces were measured and recorded preoperatively and postoperatively. Preoperative and postoperative values of the visual analog scale (VAS) and the Oxford knee score were recorded at regular intervals of two months. The surgical procedure (a PFO) was performed after obtaining informed written consent and getting anesthetic fitness for the procedure.

Data were entered into Excel and analyzed using IBM SPSS Statistics for Windows, Version 20.0. (IBM Corp., Armonk, NY).

## Results

Descriptive statistics are provided in Table 1.

**Table 1:** Descriptive statistics. SD, standard deviation; VAS, visual analog scale.

Variables (n = 60)		Mean ± SD or Frequency	p-value
Age (years)		51.8 ± 4.1	
Gender	Male	16 (26.7%)	
	Female	44 (73.3%)	
Duration of surgery (minutes)		23.80 ± 3.05	
Medial joint space (mm)	Preoperative	1.45 ± 0.28	< 0.00000001
	Postoperative	4.63 ± 0.668	
Lateral joint space (mm)	Preoperative	8.86 ± 1.27	< 0.00000001
	Postoperative	4.72 ± 0.799	
VAS	Preoperative	7.90 ± 0.79	< 0.00000001
	Postoperative	2.32 ± 0.792	
Oxford knee score	Preoperative	20.82 ± 1.97	< 0.00000001
	Postoperative	35.92 ± 3.509	
Complication	No complications	54 (90.0%)	
	Peroneal nerve palsy	0 (00.0%)	
	Loss of dorsiflexion of the great toe	3 (5.0%)	
	Sensory loss over the dorsum of the foot	0 (00.0%)	
	Superficial wound infection	3 (5.0%)	
	Deep wound infection	0 (00.0%)	

A total of 60 patients were enrolled in the study: 16 (26.7%) were men, and 44 (73.3%) were women. The mean age of patients was 51.8 ± 4.1 years. The total duration of surgery from incision to closure of the wound was 23.80 ± 3.05 minutes. Preoperative mean medial joint space measurement on standard anteroposterior radiograph was 1.45 ± 0.28 mm. The mean preoperative lateral joint space was 8.86 ± 1.27 mm. The mean preoperative VAS for pain measurement was 7.90 ± 0.79. The recorded mean preoperative Oxford knee score was 20.82 ± 1.97. Recorded levels of mean postoperative medial joint space improved to 4.63 ± 0.668 mm, and mean postoperative lateral joint space was 4.72 ± 0.79 mm.

Mean recorded levels of VAS for pain postoperatively were 2.32 ± 0.792, which improved significantly from 7.90 ± 0.79. The mean recorded level of the postoperative Oxford knee score was 35.92 ± 3.509, which improved significantly in comparison to preoperative levels. The majority of patients (n=56) showed no complications. None of the patients developed peroneal nerve injury. There was a loss of dorsiflexion of the great toe in three patients, which resolved spontaneously. In three patients, there was superficial wound infection, which resolved with antibiotics and daily dressing.

## Discussion

Proximal fibular osteotomy is the new treatment option for medial compartment knee joint OA. Although high tibial osteotomy and unicompartmental arthroplasty were previously the treatment options for medial compartmental arthritis, both have their advantages and disadvantages and are associated with major complications [14], which include infection, deep vein thrombosis (DVT), insufficient correction, intra-articular fractures, peroneal nerve injury, compartment syndrome, and knee stiffness. In contrast, late complications of this procedure include delayed union or nonunion, deformity recurrence, and internal fixation failure [15]. There is still insufficient and inconclusive evidence in the literature on PFO in medial compartment OA of the knee. A correctly performed fibular osteotomy (in terms of accurate height from the fibular head, the length of the fibular chunk removed, and peroneal nerve protection) is paramount for a good outcome. Performing fibular osteotomy at the level of about 4-7 cm distal to fibular head decreases the risk of peroneal nerve injury [16]. Yang et al. performed a study on 110 patients with medial compartment arthritis that were followed for over two years [1]. In this study, the preoperative Knee Society Score was 45 ± 21.3, while postoperatively, it was 92.3 ± 31.7.

Additionally, the mean VAS score preoperatively was seven, which significantly decreased to two in the postoperative period. Based on these results, there was a significant improvement in the function of the knee and relieving pain [1]. In a study conducted by Wang et al. on PFO for medial compartment, OA pain relief was observed in all patients after PFO; the mean VAS scores improved dramatically from 8.02 ± 1.50 preoperatively to 2.74 ± 2.34 postoperatively [17].

In another study on 30 patients who were treated by PFO, the mean VAS improved significantly from 6.9 to 2.1 (p < 0.005). The average preoperative Oxford score also showed a significant improvement from 52.2 preoperatively to 79 in the postoperative period (p < 0.005). There was an improvement in the medial joint space from 1.3 ± 0.8 mm preoperatively to 4.2 ± 2.7 mm in the postoperative period [18].

In our study, in terms of improvement, two significant differences were found. When we compared a mean preoperative medial joint space that initially was 1.45 ± 0.28 mm, it improved significantly to 4.63 ± 0.668 mm, and lateral joint space changed from 8.86 ± 1.27 mm to 4.72 ± 0.79 mm. In terms of pain relief, the mean VAS before surgery was 7.90 ± 0.79, which improved to 2.32 ± 0.792; that was significant in terms of patient relief of pain. Similarly, in terms of comparison of function, the mean preoperative Oxford score was 20.82 ± 1.97. Postoperatively it improved to 35.92 ± 3.509. All these findings, i.e., changes in medial joint space, lateral joint space, VAS, and Oxford knee score, were all suggestive of the beneficial effects of PFO in terms of pain relief and function. The results in our study were comparable to the studies conducted by other authors such as Yang et al. [1], Wang et al. [17], and Subash and Naidu [18]. PFO is not a new procedure, surgeons still are reluctant to adopt a procedure for medial compartment OA. Studies with longer follow up time are still required to evaluate the effectiveness of this procedure thoroughly.

## Conclusions

Proximal fibular osteotomy is a simple, safe, less time consuming, and effective procedure for pain relief and functional recovery. It requires little rehabilitation and is associated with little or no complications. After a review of the results of our study it was revealed that this procedure is reasonably good both clinically and radiologically, and can be recommended for medial compartment OA of the knee joint.

## References

[1] Yang Z-Y, Chen W, Li C-X (2015). Medial compartment decompression by fibular osteotomy to treat medial compartment knee osteoarthritis: a pilot study. Orthopedics.

[2] Cross M, Smith E, Hoy D (2014). The global burden of hip and knee osteoarthritis: estimates from the Global Burden of Disease 2010 study. Ann Rheum Dis.

[3] Pavelka K, Coste P, Géher P, Krejci G (2010). Efficacy and safety of piascledine 300 versus chondroitin sulfate in a 6 months treatment plus 2 months observation in patients with osteoarthritis of the knee. Clin Rheumatol.

[4] Kon E, Filardo G, Drobnic M, Madry H, Jelic M, van Dijk N, Della Villa S (2012). Non-surgical management of early knee osteoarthritis. Knee Surgery Sports Traumatol Arthrosc.

[5] Michael JW, Schlüter-Brust KU, Eysel P (2010). The epidemiology, etiology, diagnosis, and treatment of osteoarthritis of the knee. Dtsch Aerzteblatt Online.

[6] Page CJ, Hinman RS, Bennell KL (2011). Physiotherapy management of knee osteoarthritis. Int J Rheum Dis.

[7] Seed SM, Dunican KC, Lynch AM (2011). Treatment options for osteoarthritis: considerations for older adults. Hosp Pract.

[8] Sprenger TR, Doerzbacher JF (2003). Tibial osteotomy for the treatment of varus gonarthrosis. Survival and failure analysis to twenty-two years. J Bone Joint Surg Am.

[9] Aglietti P, Buzzi R, Vena LM, Baldini A, Mondaini A (2003). High tibial valgus osteotomy for medial gonarthrosis: a 10- to 21-year study. J Knee Surg.

[10] Hanssen AD, Stuart MJ, Scott RD, Scuderi GR (2001). Surgical options for the middle-aged patient with osteoarthritis of the knee joint. Instr Course Lect.

[11] Schnurr C, Jarrous M, Güdden I, Eysel P, König DP (2013). Preoperative arthritis severity as a predictor for total knee arthroplasty patients’ satisfaction. Int Orthop.

[12] Wu L, Hahne H-J, Hassenpflug J (2004). [A long-term follow-up study of high tibial osteotomy in medial compartment osteoarthrosis]. Zhonghua Wai Ke Za Zhi.

[13] Giagounidis EM, Sell S (1999). High tibial osteotomy: factors influencing the duration of satisfactory function. Arch Orthop Trauma Surg.

[14] Hofmann S, Lobenhoffer P, Staubli A, Van Heerwaarden R (2009). Osteotomien am kniegelenk bei monokompartmentarthrose [Osteotomies of the knee joint in patients with monocompartmental arthritis]. Orthopade.

[15] Portner O (2014). High tibial valgus osteotomy: closing, opening or combined? Patellar height as a determining factor. Clin Orthop Relat Res.

[16] Chen H-W, Liu G-D, Ou S, Zhao G-S, Pan J, Wu L-J (2014). Open reduction and internal fixation of posterolateral tibial plateau fractures through fibula osteotomy-free posterolateral approach. J Orthop Trauma.

[17] Wang X, Wei L, Lv Z (2017). Proximal fibular osteotomy: a new surgery for pain relief and improvement of joint function in patients with knee osteoarthritis. J Int Med Res.

[18] Subash DY, Naidu DGK (2018). The role of proximal fibular osteotomy in the management of medial compartment osteoarthritis of the knee. Int J Orthop Sci.

